# Investigation of Newly Diagnosed Drug-Naive Patients with Systemic Autoimmune Diseases Revealed the Cleaved Peptide Tyrosine Tyrosine (PYY 3-36) as a Specific Plasma Biomarker of Rheumatoid Arthritis

**DOI:** 10.1155/2021/5523582

**Published:** 2021-06-17

**Authors:** Jozsef A. Balog, Agnes Kemeny, Laszlo G. Puskas, Szilard Burcsar, Attila Balog, Gabor J. Szebeni

**Affiliations:** ^1^Biological Research Centre, Szeged, Hungary; ^2^PhD School of Biology, University of Szeged, Szeged, Hungary; ^3^Department of Medical Biology, University of Pecs, Pecs, Hungary; ^4^Department of Pharmacology and Pharmacotherapy, Medical School, University of Pecs, Pecs, Hungary; ^5^Avicor Ltd. Szeged, Hungary; ^6^Avidin Ltd., Szeged, Hungary; ^7^Department of Rheumatology and Immunology, Faculty of Medicine, Albert Szent-Gyorgyi Health Centre, University of Szeged, Szeged, Hungary; ^8^Department of Physiology, Anatomy and Neuroscience, Faculty of Science and Informatics, University of Szeged, Szeged, Hungary; ^9^CS-Smartlab Devices, Kozarmisleny, Hungary

## Abstract

There is a current imperative to reveal more precisely the molecular pathways of early onset of systemic autoimmune diseases (SADs). The investigation of newly diagnosed drug-naive SAD patients might contribute to identify novel disease-specific and prognostic markers. The multiplex analysis of 30 plasma proteins in 60 newly diagnosed drug-naive SADs, such as RA (rheumatoid arthritis, *n* = 31), SLE (systemic lupus erythematosus, *n* = 19), and SSc (systemic scleroderma, *n* = 10) patients, versus healthy controls (HCs, *n* = 40) was addressed. Thirty plasma cytokines were quantified using the Procarta Plex™ panel. The higher expression of IL-12p40, IL-10, IL-13, IFN-*γ*, M-CSF, IL-4, NTproBNP, IL-17A, BMP-9, PYY (3-36), GITRL, MMP-12, and TNFRSF6 was associated with RA; IL-12p40, M-CSF, IL-4, GITRL, and NTproBNP were higher in SLE; or NTproBNP, PYY (3-36), and MMP-12 were increased in SSc over HCs, respectively. The cleaved peptide tyrosine tyrosine (PYY 3-36) was elevated in RA (361.6 ± 47.7 pg/ml) vs. HCs (163.96 ± 14.5 pg/ml, mean ± SEM, ^∗∗∗^*p* = 4 × 10^−5^). The CI (95%) was 268.05-455.16 pg/ml for RA vs. 135.55-192.37 pg/ml for HCs. The elevated PYY (3-36) level correlated significantly with the increased IL-4 or GITRL concentration but not with the clinical scores (DAS28, CRP, ESR, RF, aMCV). We are the first to report cleaved PYY (3-36) as a specific plasma marker of therapy-naive RA. Additionally, the multiplex plasma protein analysis supported a disease-specific cytokine pattern in RA, SLE, and SSc, respectively.

## 1. Introduction

Systemic autoimmune rheumatic diseases (SADs) including rheumatoid arthritis (RA), systemic lupus erythematosus (SLE), and systemic sclerosis (SSc) are characterized by an abnormal immune system response, complement dysregulation, imbalance of cytokines production, and inflammation [1]. Their etiology, complex pathogenesis, heterogeneous presentation, and unpredictable disease course are still not fully understood [2]. Therefore, the limitation in diagnosing, classifying, and treating both RA, SLE, and SSc is significant. Clinical remission is reached in less than half of the patients, the personalized therapeutic strategy is still lacking, and the gap between the first symptoms and the diagnosis may often lead to irreversible pathologic changes [3]. SADs display clinical heterogeneity as presented by variable prognosis, unpredictable susceptibility to rapid progression to structural damage in joints in RA, and severe extra-articular organ manifestations in SLE and SSc. In summary, the need for biomarkers facilitating early diagnosis and profiling those individuals at the highest risk for a poor outcome has become essential [4]. Biomarkers of RA [5, 6], SLE [7, 8], or SSc [9, 10] have been recently reviewed elsewhere. Many of the previous studies have been performed in established or late-stage disease in SADs. There is a current imperative to reveal more precisely the molecular pathways of early treatment-naive SADs [11]. Furthermore, very few studies have reported a systematic molecular characterization in RA, SLE, and SSc parallelly, but none in early treatment-naive patients with SADs. The investigation of newly diagnosed drug-naive SAD patients might contribute to identify novel disease-specific and prognostic markers. The parallel investigation of SADs also could give us the possibility to recognize novel checkpoints in their pathways and unknown molecular therapeutic targets. Therefore, we aimed to assay the plasma content of thirty soluble mediators in newly diagnosed therapy-naive RA, SLE, or SSc patients versus age- and gender-matched healthy controls.

## 2. Materials and Methods

### 2.1. Patient and Public Involvement

Patients were enrolled during regular visits at the Department of Rheumatology and Immunology (University of Szeged). Healthy controls were voluntary staff members of the BRC or University of Szeged. Subjects were informed about the research by a physician. Written informed consent was obtained from all subjects, and our study was reviewed and approved by an independent ethical committee of the university. Laboratory studies and interpretations were performed on coded samples lacking personal and diagnostic identifiers. The study adhered to the tenets of the most recent revision of the Declaration of Helsinki. Details about the study design and handling of biological materials were submitted to the Human Investigation Review Board of the University of Szeged under the 149/2019-SZTE Project Identification code.

### 2.2. Study Cohorts

The multiplex protein analysis of 60 drug-naive SAD patients, RA patients (*n* = 31, age median 57, 70.9% female (F), Supplementary Table 1); SLE patients (*n* = 19, age median 51, 89.4% F, Supplementary Table 2); SSc patients (*n* = 10, age median 51, 88.9% F, Supplementary Table 3), and 40 age- and gender-matched healthy controls (age median 48.5, 72.5% F) was performed. We enrolled newly diagnosed drug-naive RA, SLE, and SSc patients, who had not received antirheumatic treatment, including nonsteroidal anti-inflammatory drugs (NSAIDs), disease-modifying antirheumatic drugs (DMARDs), or glucocorticoids until the time of blood sampling. The RA patients were diagnosed according to the latest ACR/EULAR criteria [12] (Supplementary Table 1). The SLE patients who met the 2012 Systemic Lupus Collaborating Clinics (SLICC) criteria and in whom active, newly diagnosed SLE was present were considered eligible [13]. Several clinical and immunoserological parameters were present at the time of diagnosis of SLE (Supplementary Table 2). Ten newly diagnosed patients fulfilling the criteria proposed by the 2013 ACR/EULAR classification criteria for SSc were enrolled [14]. Two out of ten were further classified as those with limited cutaneous SSc, and eight out of ten with diffuse cutaneous scleroderma according to LeRoy et al. [15] (Supplementary Table 3). Healthy controls were age and gender matched to the patients, having a negative history of rheumatic symptoms and negative status upon detailed physical and laboratory examination. No comorbidities were detected in patients and controls that could have influenced our investigation, nor did they take any medication that could have interfered with the measurements.

### 2.3. Measurement of Plasma Proteins

After the withdrawal of 20 ml blood into an EDTA vacutainer (Becton Dickinson), human peripheral blood mononuclear cells and plasma samples were purified by Leucosep tubes (Greiner Bio-One, Austria). PBMCs were used for immunophenotyping in another project. Plasma fractions were stored at -80°C in aliquots before running the assay. Luminex xMAP technology was used to determine the protein concentrations of 30 distinct cytokines/chemokines performing Procarta Plex™ Immunoassay (ThermoFisher Scientific, Waltham, MA, USA) according to the instructions of the manufacturer. The Luminex panel was designed by the authors quantifying the proteins listed in Supplementary Table 4. Briefly, all samples were thawed and diluted with sterile phosphate-buffered saline (PBS) to 1 : 1 and were tested in a blind fashion and in duplicate. 25 *μ*l volume of each sample, standard, and universal assay buffer was added to a 96-well plate (provided with the kit) containing 50 *μ*l of capture antibody-coated, fluorescent-coded beads. Biotinylated detection antibody mixture and streptavidin-PE were added to the plate after the appropriate incubation period. After the last washing step, 120 *μ*l reading buffer was added to the wells, and the plate was incubated for an additional 5 minutes and read on the Luminex MAGPIX® instrument. Luminex xPonent 4.2 software was used for data acquisition. Five-PL regression curves were generated to plot the standard curves for all analytes by the Analyst 5.1 (Merck Millipore, Darmstadt, Germany) software calculating with bead median fluorescence intensity values. The panel of the investigated 30 plasma proteins and the range of the detection (in pg/ml from the lower limit to the upper limit) are available in the Supplementary Table 4. Data were pooled from two independent measurements and plotted in GraphPad Prism.

### 2.4. Statistical Analysis

The arithmetic mean (mean), standard deviation (SD), and the standard error of the mean (SEM) of the plasma cytokine concentrations were calculated. The pairwise comparison of the concentrations of each cytokine of patients versus healthy controls (RA vs. HC; SLE vs. HC; SSc vs. HC) was carried out by one-way ANOVA (^∗^*p* < 0.05; ^∗∗^*p* < 0.01, ^∗∗∗^*p* < 0.001). The 95% confidence intervals (CI, 95%) were calculated between patients and HCs for each cytokine separately. Calculations were done in Microsoft Excel.

## 3. Results and Discussion

**Figure 1:**
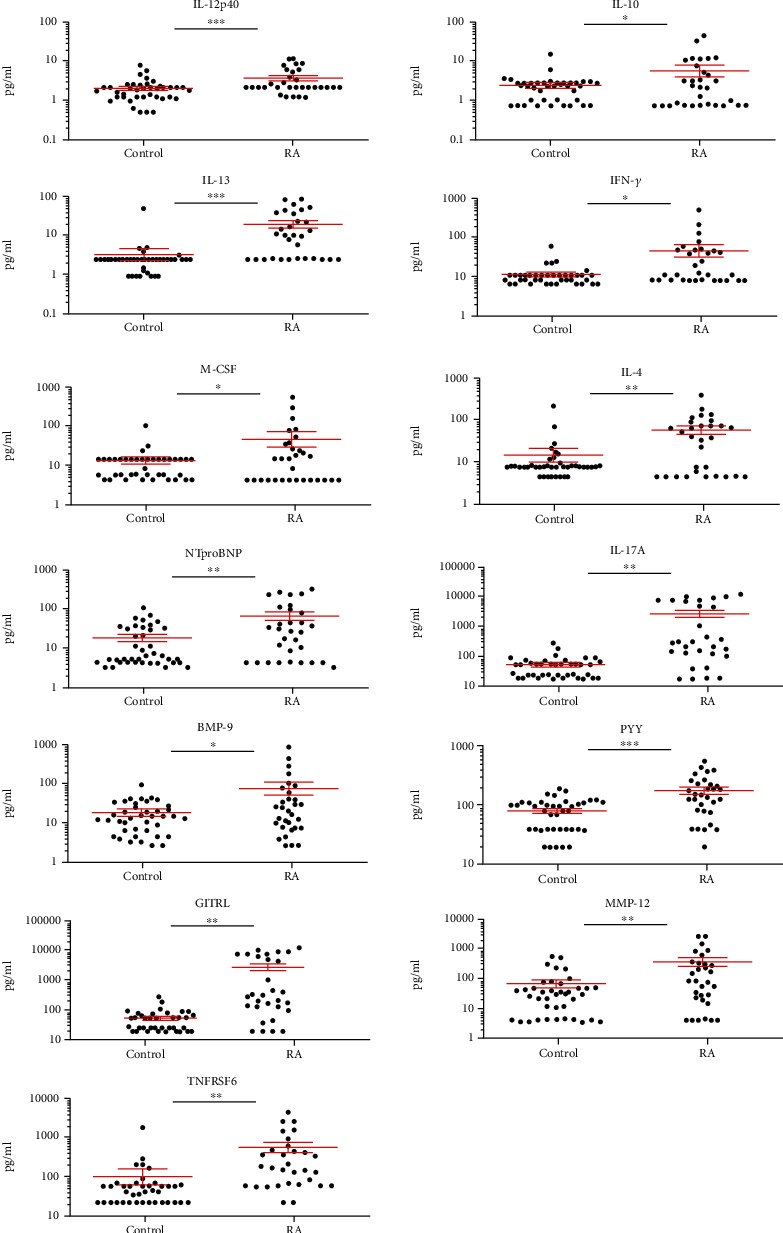
The scatter plots of the protein concentrations of plasma proteins (pg/ml) in drug-naive RA (*n* = 31) patients versus age- and gender-matched healthy controls (*n* = 40) with significant differences (one-way ANOVA, ^∗^*p* < 0.05; ^∗∗^*p* < 0.01, ^∗∗∗^*p* < 0.001) measured by the Luminex MAGPIX technology. The cleaved peptide tyrosine tyrosine PYY (3-36) was detected as being a novel marker of early-onset therapy-naive rheumatoid arthritis (RA) (^∗∗∗^*p* = 4 × 10^−5^). The arithmetic mean ± SEM are demonstrated for each cytokine (pg/ml) of RA (*n* = 31) patients versus age- and gender-matched healthy controls (*n* = 40). One dot is the average of two technical replicates.

The following 30 plasma cytokines in the custom Procarta Plex™ panel were quantified in the RA, SLE, and SSc patients and healthy controls (HCs): SDF-1a, GITRL, IL-1b, IL-2, IL-4, IL-5, IL-33, IL-10, Insulin, PYY (3-36), CCL22, IL-13, IL-17A, Gal-3, FKN, IFN-*γ*, GM-CSF, Leptin, MMP-12, NTproBNP, MCP-1, APRIL, TNFRSF6, BDNF, BMP-9, IL-12p40, BAFF, M-CSF, Survivin, and CD40-ligand (Supplementary Table 4). These markers under investigation were selected by the authors based on preliminary experiments and literature data. Thirteen cytokines were significantly elevated in RA vs. HCs (Figure 1); the concentrations of eleven cytokines in RA patients showed nonoverlapping confidence interval with HCs (Table 1).

The protein concentrations were the following (RA *vs.* HC, mean ± SEM, respectively), IL-12p40: 8.90 ± 1.16*vs.*4.18 ± 0.45 pg/ml; IL-13: 41.74 ± 8.93*vs.*7.25 ± 2.46 pg/ml; IFN-*γ*: 98.83 ± 34.31*vs.*23.64 ± 2.9 pg/ml; IL-4: 121.79 ± 28.21*vs.*31.14 ± 10.98 pg/ml; NTproBNT: 142.07 ± 34.96*vs.*39.24 ± 7.44 pg/ml; IL-17A: 154.17 ± 51.57*vs.*15.98 ± 4.27 pg/ml; BMP-9: 160.67 ± 62.45*vs.*37.78 ± 5.46 pg/ml; PYY (3-36): 361.6 ± 47.73*vs.*163.96 ± 14.5 pg/ml; GITRL: 901.22 ± 269.22*vs.*110.19 ± 15.96 pg/ml; MMP-12: 833.00 ± 273.93*vs.*154.26 ± 43.99 pg/ml; and TNFRSF6: 1200.63 ± 358.90*vs.*209.45 ± 90.77 pg/ml (Table 1 and Figure 1).

**Figure 2:**
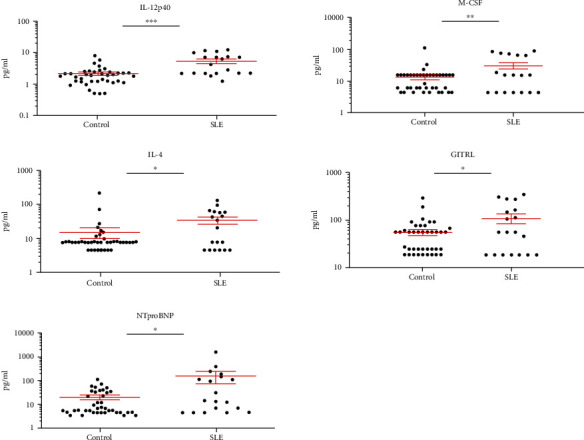
The scatter plots of the protein concentrations (pg/ml) of SLE (*n* = 19) patients versus age- and gender-matched healthy controls (*n* = 40) with significant differences (one-way ANOVA, ^∗^*p* < 0.05; ^∗∗^*p* < 0.01, ^∗∗∗^*p* < 0.001). One dot is the average of two technical replicates.

Five cytokines, IL-12p40, M-CSF, IL-4, GITRL, and NTproBNP, were significantly elevated in SLE *vs.* HC (Figure 2), and only IL-12p40 had nonoverlapping CI with HC: 10.86 ± 1.7*vs.*4.18 ± 0.45 pg/ml (Table 1).

**Figure 3:**
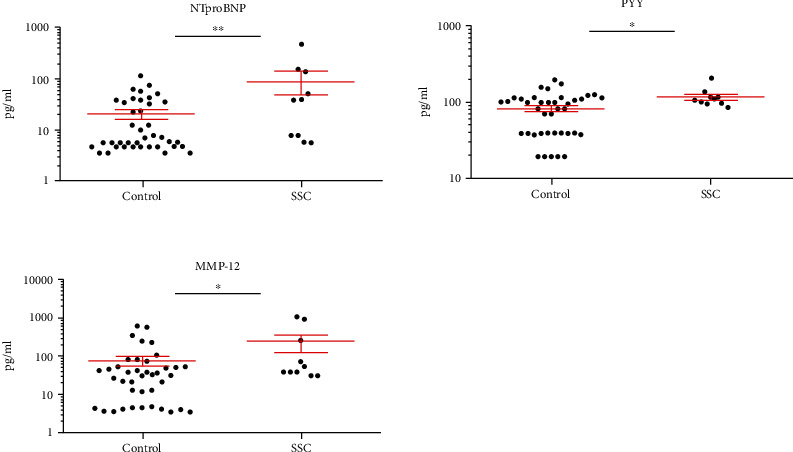
The scatter plots of the protein concentrations (pg/ml) of SSc (*n* = 10) patients versus age- and gender-matched healthy controls (*n* = 40) with significant differences (one-way ANOVA, ^∗^*p* < 0.05; ^∗∗^*p* < 0.01, ^∗∗∗^*p* < 0.001). One dot is the average of two technical replicates.

Three cytokines, NTproBNP, PYY (3-36), and MMP-12, were significantly increased in SSc *vs.* HC (Figure 3), but all had overlapping CI (Table 1).

We are the first to report the cleaved peptide tyrosine tyrosine PYY (3-36) is an early marker of drug-naive RA *vs.* HC. The margin of errors (ME) of the CI are 93.55 for PYY in the RA (*n* = 31) group and 28.41 for HC (*n* = 40), the 95% CI falls between 268.05 and 455.16 pg/ml (361.6 pg/ml mean ± 93.55 ME) for RA, and the 95% CI is between 135.55 and 192.37 pg/ml for HC (163.96 pg/ml mean ± 28.41 ME) (Table 1). Comparing the concentrations (mean ± SEM) of PYY (3-36) in RA (361.6 ± 47.7 pg/ml) *vs.* SLE (189.3 ± 27.5 pg/ml), it was significantly higher in RA (^∗^*p* = 1.07 × 10^−2^), and the CI is not overlapping; 95% CI falls between 268.05 and 455.16 pg/ml for RA and 135.4 and 243.3 pg/ml for SLE. The amount of the PYY (3-36) had no significant difference (mean ± SEM) between RA (361.6 ± 47.7 pg/ml) *vs.* SSC (232.9 ± 21.5 pg/ml) with CI for RA (268.05-455.16 pg/ml, 95%) and SSC (190.73-275.18 pg/ml, 95%). The amount of the PYY (3-36) had no significant difference (mean ± SEM) between SLE (189.3 ± 27.5 pg/ml) and SSC (232.9 ± 21.5 pg/ml) with CI for SLE (135.4-243.3 pg/ml, 95%) and SSC (190.73-275.18 pg/ml, 95%). There was no difference in the concentrations of the following cytokines in either RA, SLE, or SSc patients versus HCs: SDF-1a, IL-1b, IL-2, IL-5, IL-33, Insulin, CCL22, Gal-3, FKN, GM-CSF, Leptin, MCP-1, APRIL, BDNF, BAFF, Survivin, and CD40-ligand, respectively.

**Figure 4:**
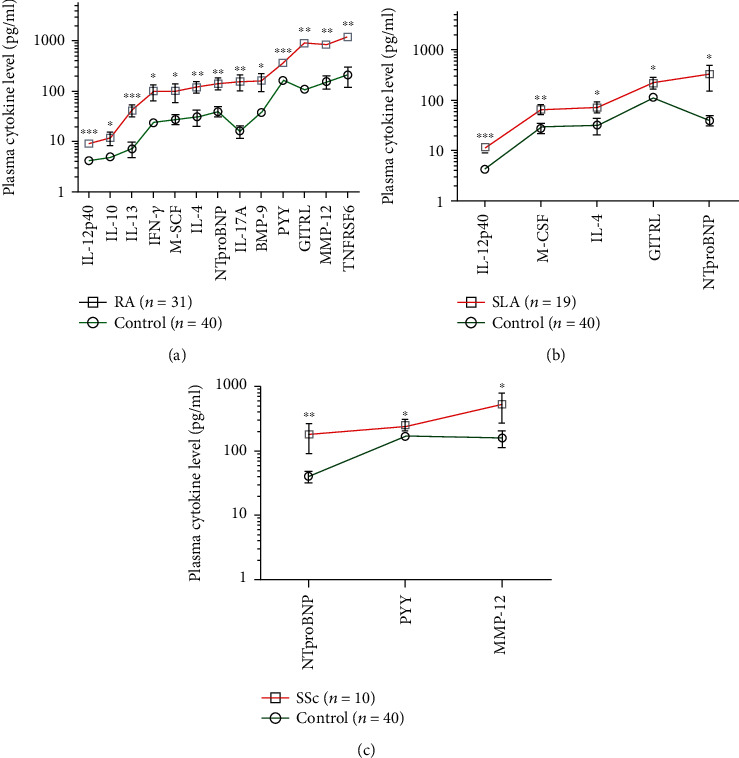
The concentrations of plasma cytokines (pg/ml) in the ascending order of (a) RA (*n* = 31), (b) SLE (*n* = 19), and (c) SSc (*n* = 10) patients versus age- and gender-matched healthy controls (*n* = 40) measured by the Luminex MAGPIX technology. The cleaved peptide tyrosine tyrosine PYY (3-36) is a novel marker of early-onset therapy-naive rheumatoid arthritis (RA) (^∗∗∗^*p* = 4 × 10^−5^). The arithmetic mean ± SEM are demonstrated for each cytokine on a log10 scale in GraphPad Prism software. The connecting lines delineate a characteristic pattern of proteins measured specific to drug-naive autoimmune diseases (red) such as (a) RA, (b) SLE, and (c) SSc or healthy controls (green).

Thirteen cytokines showed significantly elevated concentrations in the plasma of SAD patients, distinguishing RA (Figure 1), SLE (Figure 2), or SSc (Figure 3) from HCs, respectively. Arranging the values in an ascending order of the concentrations of the plasma cytokines as a prototype model delineates a characteristic cytokine/chemokine pattern of RA, SLE, and SSc. Therefore, after performing the assay, we propose a simple diagnostic algorithm fitting the curves of cytokine concentration values of early drug-naive SADs patients *vs.* HCs to diagnose drug-naïve RA, SLE, or SSc (Figure 4). Based on these data, we propose the machine-learning automated classification of drug-naïve RA, SLE, and SSc, which should be further verified in a dedicated clinical study.

Better identification of the specific molecular players of the early stage of SADs may contribute to the recognition of novel prognostic markers and could facilitate the pathogenesis-appropriate timing of therapeutic interventions. In summary, we have quantified plasma proteins in early SAD patients, prior to therapeutic modification of the disease pathology. A characteristic pattern of soluble mediators was revealed distinguishing early diagnosed therapy-naive RA, SLE, or SSc from HCs. These eleven markers with nonoverlapping CI (95%) were associated with RA: IL-12p40, IL-10, IL-13, IFN-*γ*, M-CSF, IL-4, NTproBNP, IL-17A, BMP-9, PYY, GITRL, MMP12, and TNFRSF6, and one marker, IL-12p40, with SLE versus HCs (Table 1). However, we suggest our Luminex panel for in vitro diagnostics and the development of a simple algorithm to differentiate therapy-naive RA, SLE, or SSc based on the profile of protein concentrations (Figure 4).

There were no significant correlations in PYY (3-36) concentration (pg/ml values) compared to CRP, ESR, RF, aMCV, and DAS28 scores in RA, separately. However, the concentration of two cytokines in the plasma of drug-naive RA patients, the IL-4 or GITRL concentrations, showed correlation with bioactive PYY (3-36) level, respectively. (Supplementary Figure 1). The elevated GITRL or IL-4 have been thoroughly studied in RA and associated with disease severity linked to Th17-cell activation or autoantibody induction, respectively [16, 17]. However, their association with the increased concentration with PYY (3-36) needs further mechanistic studies to explain. We describe here the cleaved peptide tyrosine tyrosine (PYY 3-36) as a plasma marker of early-onset drug-naive RA. The 1-36 peptide YY (PYY) as a gut hormone has been reported to be activated by dipeptidyl peptidase-IV (DPP-IV or CD26) cleavage resulting in PYY (3-36) which binds to Y2 (coded by *NPY2R*) receptors in the hippocampus reducing appetite [18, 19]. Chen et al. showed that plasma PYY concentration was negatively correlated with the increase of body weight in RA patients followed by etanercept therapy [20]. The authors have no direct evidence, but the literature and our data may suggest that the elevated plasma PYY (3-36) level contributes to the reduced appetite and cachexia of RA patients. Chen et al. already shed light on PYY as a link between the gastrointestinal neuroendocrine axis and the immune system [21]. The possible role of Y2 receptor + microglia, monocytes/macrophages, granulocytes, and lymphocytes on the immune homeostasis and regulation of inflammation has been recently reviewed [22]. Further research is needed to ascertain the role of PYY (3-36) in early-onset RA and its possible effect on the innate or adaptive arm of the immune system and whether it has a regulatory effect or its increase in the blood is just a consequence of the pathomechanism of RA.

## 4. Conclusions

We are the first to report PYY (3-36) as a specific plasma marker of drug-naive RA. Additionally, the multiplex analysis of 30 plasma proteins supported a disease-specific cytokine pattern in RA, SLE, and SSc, respectively. Based on these data, we could delineate a prototype model for the machine-learning automated classification of drug-naive RA, SLE, and SSc.

## Figures and Tables

**Table 1:** The summary of the significant differences of plasma cytokine concentrations between drug-naive autoimmune patients (RA, SLE, SSc) and healthy controls (HCs) in a pairwise comparison. The arithmetic means (mean), standard deviation (SD), and the standard error of the mean (SEM) of the plasma cytokine concentrations were calculated. The pairwise comparison of the concentrations of each cytokine of patients versus healthy controls (RA vs. HC; SLE vs. HC; SSc vs. HC) was carried out by one-way ANOVA (^∗^*p* < 0.05; ^∗∗^*p* < 0.01, ^∗∗∗^*p* < 0.001). The 95% confidence intervals (CI, 95%) were calculated between patients and HCs for each cytokine separately. Italic emphasis corresponds to overlapping patient's CI with HCs.

Cytokine	Cohort	Mean (pg/ml)	SD	SEM	One-way ANOVA (*p*)	CI (95%)
IL-12p40	HC	4.18	2.85	0.45	9.7*E*-05	3.30-5.06
	RA	8.90	6.45	1.16		6.63-11.17

IL-10	HC	4.96	4.66	0.74	3.2*E*-02	*3.51-6.40*
	RA	12.02	19.69	3.54		*5.09-18.95*

IL-13	HC	7.25	15.53	2.46	9.7*E*-05	2.43-12.06
	RA	41.74	49.74	8.93		24.23-59.25

IFN-*γ*	HC	23.64	18.35	2.90	1.6*E*-02	17.96-29.33
	RA	98.83	191.03	34.31		31.58-166.07

M-CSF	HC	27.34	34.09	5.39	4.8*E*-02	*16.78-37.91*
	RA	100.58	227.01	40.77		*20.66-180.49*

IL-4	HC	31.14	69.44	10.98	1.7*E*-03	9.62-52.66
	RA	121.79	157.07	28.21		66.50-177.09

NTproBNP	HC	39.24	47.07	7.44		24.64-53.82
	RA	142.07	194.66	34.96	1.9*E*-03	73.55-210.59

IL-17A	HC	15.98	26.99	4.27	3.5*E*-03	7.62-24.34
	RA	154.17	288.13	51.75		52.74-255.6

BMP-9	HC	37.78	34.56	5.46	2.9*E*-02	27.07-48.49
	RA	160.67	347.69	62.45		38.27-283.06

PYY	HC	163.96	91.68	14.50	4.0*E*-05	135.55-192.37
	RA	361.60	265.77	47.73		268.05-455.16

GITRL	HC	110.19	100.94	15.96	1.4*E*-03	78.91-141.47
	RA	901.22	1498.96	269.22		373.54-1428.89

MMP-12	HC	154.26	278.20	43.99	7.4*E*-03	68.05-240.48
	RA	833.00	1525.20	273.93		296.09-1369.91

TNFRSF6	HC	209.45	574.09	90.77	3.9*E*-03	31.54-387.36
	RA	1200.63	1998.27	358.90		497.18-1904.07

IL-12p40	HC	4.18	2.85	0.45	5.7*E*-06	3.29-5.06
	SLE	10.86	7.43	1.70		7.52-14.20

M-CSF	HC	27.34	34.09	5.39	6.8*E*-03	*16.77-37.90*
	SLE	63.06	63.94	14.67		*34.31-91.81*

IL-4	HC	31.14	69.44	10.98	5.2*E*-02	*9.61-52.65*
	SLE	70.16	73.03	16.75		*37.32-102.99*

GITRL	HC	110.19	100.94	15.96	1.3*E*-02	*78.90-141.47*
	SLE	217.58	222.56	51.06		*117.50-317.65*

NTproBNP	HC	39.24	47.07	7.44	1.9*E*-02	*24.64-53.82*
	SLE	315.89	727.91	166.99		*1.69-643.19*

NTproBNP	HC	39.235	47.07	7.44	3.8*E*-03	*24.64-53.82*
	SSc	174.206	272.76	86.25		*5.15-343.26*

PYY	HC	163.9605	91.68	14.50	3.1*E*-02	*135.54-192. 37*
	SSc	232.962	68.12	21.54		*190.73-275.18*

MMP-12	HC	154.2605	278.20	43.99	2.1*E*-02	*68.04-240.47*
	SSc	514.748	798.52	252.52		*19.82-1009.66*

## Data Availability

Additional data are in the Supplementary Files, or raw data can be requested from the corresponding author.

## Supplementary Materials

Supplementary 1 Supplementary Table 1: clinical characteristics of RA study participants. The median DAS28 disease activity score was 6.01, and Q1-Q3 interquartiles were 5.4-6.5. Several clinical and immunoserological parameters were present at the time of diagnosis of RA including RF: rheumatoid factor; MCV: mutated citrullinated vimentin; CRP: C-reactive protein; ESR: erythrocyte sedimentation rate. Data are expressed as median and interquartile range (Q1, Q3) for continuous variables and as number (*n*) and (%) for categorical variables. BLD: below the detection limit.

Supplementary 2 Supplementary Table 2: clinical characteristics of SLE study participants. The median SLEDAI-2K activity score was 16, and Q1-Q3 interquartiles were 10-21. Several clinical and immunoserological parameters were present at the time of diagnosis of SLE including ANA (antinuclear antibodies); anti-DNA antibody; LA (lupus anticoagulant) (activated partial thromboplastin time > 40 s, 8/19); hemolytic anemia (hematocrit < 0.35, 4/19); nonhemolytic anemia (hematocrit < 0.35, 4/19); leukopenia (leukocyte count < 3000/mm^3^, 8/19); lymphopenia (lymphocyte count < 1500/mm^3^, 7/19); and thrombocytopenia (thrombocyte count < 100000/×10^6^ l, 7/19). Data are expressed as median and interquartile range (Q1, Q3) for continuous variables and as number (*n*) and (%) for categorical variables.

Supplementary 3 Supplementary Table 3: clinical characteristics of SSc study participants. Several clinical and immunoserological parameters were present at the time of diagnosis of SSc including ANA (antinuclear antibodies); anti-Scl-70, anti-Scl-70 antibodies; ACA (anticentromere antibodies); and anti-RNA polymerase III antibodies. Data are expressed as median and interquartile range (Q1, Q3) for continuous variables and as number (*n*) and (%) for categorical variables. dcSSc: diffuse cutaneous SSc; lcSSc: limited cutaneous SSc; skin score^b^: modified Rodnan skin thickness score.

Supplementary 4 Supplementary Table 4: the list of the proteins measured in the plasma of the human subjects enrolled in the study. The lower range of the detection corresponds to the threshold of the sensitivity of the assay. The Procarta Plex™ panel was designed by the authors, and the assay was loaded on a MAGPIX Luminex instrument.

Supplementary 5 Supplementary Figure 1: the Pearson correlations of the plasma concentrations of IL-4 *vs.* PYY (3-36) or GITRL *vs.* PYY (3-36) in RA patients were analyzed in Microsoft Excel. The Pearson correlation coefficients were 0.819 for IL-4 vs. PYY (3-36) and 0.864 for GITRL vs. PYY (3-36). The *t*-statistic (*t*) was calculated in Microsoft Excel following this equation≔(*r*∗SQRT (*n* − 2))/(SQRT (1 − *r*^2^)), where *n* is number of subjects and *r* is the Pearson coefficient. Then, the *p* value was calculated in Microsoft Excel following this equation: = TDIST (*t*, DF, tails), where DF = *n* − 2, degree of freedom. The*p*was1.8 × 10^−8^ (^∗∗∗^) for the correlation of IL-4 vs. PYY (3-36) concentrations. The*p*was3.9 × 10^−10^ (^∗∗∗^) for the correlation of GITRL vs. PYY (3-36) concentrations.
