# Supplementary material for: Investigation of Newly Diagnosed Drug-Naive Patients with Systemic Autoimmune Diseases Revealed the Cleaved Peptide Tyrosine Tyrosine (PYY 3-36) as a Specific Plasma Biomarker of Rheumatoid Arthritis
Source: Mediators Inflamm. 2021 Jun 17;2021:5523582. doi: 10.1155/2021/5523582 (PMC8240466; doi:10.1155/2021/5523582)
Supplement: Supplementary 1 — Supplementary Table 1: clinical characteristics of RA study participants. The median DAS28 disease activity score was 6.01, and Q1-Q3 interquartiles were 5.4-6.5. Several clinical and immunoserological parameters were present at the time of diagnosis of RA including RF: rheumatoid factor; MCV: mutated citrullinated vimentin; CRP: C-reactive protein; ESR: erythrocyte sedimentation rate. Data are expressed as median and interquartile range (Q1, Q3) for continuous variables and as number (n) and (%) for categorical variables. BLD: below the detection limit. [file 5523582.f1.docx]

**Supplementary Table 1.** Clinical characteristics of RA study participants

| **Characteristics** | **RA patients (n=31)** | **Healthy Controls** |
| --- | --- | --- |
| Age, average ± SD, (median) | 55.5 ± 15 (57) | 47.7 ± 13.3 (48.5) |
| Gender: male/female, n, (% of female) | 9/22 (70.9) | 11/29 (72.5) |
| DAS28 activity score median (Q1, Q3) | 6.01 (5.46, 6.58) | Not applicable |
| RF (U/mL) median (Q1, Q3) | 175 (44, 500) | BLD |
| Anti-MCV (U/mL) median (Q1, Q3) | 210 (39.3, 930.5) | BLD |
| CRP (mg/L) median (Q1, Q3) | 34.6 (16.75, 57.5) | BLD |
| ESR (mm/h) median (Q1, Q3) | 51 (35.5, 72) | BLD |
