# Supplementary material for: Investigation of Newly Diagnosed Drug-Naive Patients with Systemic Autoimmune Diseases Revealed the Cleaved Peptide Tyrosine Tyrosine (PYY 3-36) as a Specific Plasma Biomarker of Rheumatoid Arthritis
Source: Mediators Inflamm. 2021 Jun 17;2021:5523582. doi: 10.1155/2021/5523582 (PMC8240466; doi:10.1155/2021/5523582)
Supplement: Supplementary 2 — Supplementary Table 2: clinical characteristics of SLE study participants. The median SLEDAI-2K activity score was 16, and Q1-Q3 interquartiles were 10-21. Several clinical and immunoserological parameters were present at the time of diagnosis of SLE including ANA (antinuclear antibodies); anti-DNA antibody; LA (lupus anticoagulant) (activated partial thromboplastin time > 40 s, 8/19); hemolytic anemia (hematocrit < 0.35, 4/19); nonhemolytic anemia (hematocrit < 0.35, 4/19); leukopenia (leukocyte count < 3000/mm3, 8/19); lymphopenia (lymphocyte count < 1500/mm3, 7/19); and thrombocytopenia (thrombocyte count < 100000/×106 l, 7/19). Data are expressed as median and interquartile range (Q1, Q3) for continuous variables and as number (n) and (%) for categorical variables. [file 5523582.f2.docx]

**Supplementary Table 2.** Clinical characteristics of SLE study participants

| **Characteristics** | **SLE patients (n=19)** | **Healthy Controls** |
| --- | --- | --- |
| Age, average ± SD, (median) | 48 ± 11.6 (51) | 47.7 ± 13.3 (48.5) |
| Gender: male/female, (% of female) | 2/17 (89.5) | 11/29 (72.5) |
| SLEDAI-2K median (Q1, Q3) | 16 (10, 21) | Not applicable |
| ANA positivity, n (%) | 17 (89.5) | 0 |
| Anti-DNA positivity, n (%) | 19 (100) | 0 |
| CRP (mg/L) median (Q1, Q3) | 5.8 (2, 19.5) | BLD |
| ESR (mm/h) median (Q1, Q3) | 44 (21, 70) | BLD |
| Low C3, C4, n (%) | 11 (57.9) | 0 |
| LA positivity, n (%) | 8 (42.1) | 0 |
| Coombs positivity, n (%) | 4 (21.1) | 0 |
| arthritis/arthralgia, n (%) | 11 (57.9) | 0 |
| photosensitivity, n (%) | 14 (73.7) | 0 |
| butterfly rash, n (%) | 5 (26.3) | 0 |
| other skin lesion, n (%) | 9 (47.4) | 0 |
| oral ulceration, n (%) | 2 (10.5) | 0 |
| pericarditis, n (%) | 3 (15.8) | 0 |
| pleuritis, n (%) | 5 (26.3) | 0 |
| Raynaud’s phenomenon, n (%) | 17 (89.5) | 0 |
| lupus nephritis, n (%) | 5 (26.3) | 0 |
| psychosis, n (%) | 2 (10.5) | 0 |
| endocarditis, n (%) | 1 (5.3) | 0 |
| interstitial lung involvement, n (%) | 2 (10.5) | 0 |
| vasculitis, n (%) | 4 (21.1) | 0 |
| lymphadenomegaly, n (%) | 6 (31.6) | 0 |
| splenomegaly, n (%) | 6 (31.6) | 0 |
| hemolytic anemia, n (%) | 4 (21.1) | 0 |
| nonhemolytic anemia, n (%) | 4 (21.1) | 0 |
| leukopenia, n (%) | 8 (42.1) | 0 |
| lymphopenia, n (%) | 7 (36.8) | 0 |
| thrombocytopenia, n (%) | 7 (36.8) | 0 |
