# Supplementary material for: Investigation of Newly Diagnosed Drug-Naive Patients with Systemic Autoimmune Diseases Revealed the Cleaved Peptide Tyrosine Tyrosine (PYY 3-36) as a Specific Plasma Biomarker of Rheumatoid Arthritis
Source: Mediators Inflamm. 2021 Jun 17;2021:5523582. doi: 10.1155/2021/5523582 (PMC8240466; doi:10.1155/2021/5523582)
Supplement: Supplementary 3 — Supplementary Table 3: clinical characteristics of SSc study participants. Several clinical and immunoserological parameters were present at the time of diagnosis of SSc including ANA (antinuclear antibodies); anti-Scl-70, anti-Scl-70 antibodies; ACA (anticentromere antibodies); and anti-RNA polymerase III antibodies. Data are expressed as median and interquartile range (Q1, Q3) for continuous variables and as number (n) and (%) for categorical variables. dcSSc: diffuse cutaneous SSc; lcSSc: limited cutaneous SSc; skin scoreb: modified Rodnan skin thickness score. [file 5523582.f3.docx]

**Supplementary Table 3.** Clinical characteristics of SSc study participants

| **Characteristics** | **SSC patients (n=10)** | **Healthy Controls** |
| --- | --- | --- |
| Age, average ± SD, (median) | 54.6 ± 10.4 (51) | 47.7 ± 13.3 (48.5) |
| Gender: male/female, (% of female) | 1/9 (88.9) | 11/29 (72.5) |
| CRP (mg/L) median (Q1, Q3) | 6.2 (4.3, 14.3) | BLD |
| ESR (mm/h) median (Q1, Q3) | 45.5 (34.25, 58.5) | BLD |
| Disease subset: lcSSc/dcSSc | 2/8 | Not applicable |
| ANA positivity, n (%) | 10 (100) | 0 |
| anti-Scl-70, n (%) | 6 (60) | 0 |
| ACA, n (%) | 2 (20) | 0 |
| anti-RNA polymerase III, n (%) | 1 (10) | 0 |
| Presence of digital ulcers, n (%) | 4 (40) | 0 |
| Raynaud phenomenon, n (%) | 10 (100) | 0 |
| Skin score^b^ median (Q1, Q3) | 19 (10.5, 20.75) | Not applicable |
| Interstitial lung disease, n (%) | 8 (80) | 0 |
| Pulmonary arterial hypertension, n (%) | 1 (10) | 0 |
| Gastrointestinal involvement, n (%) | 8 (80) | 0 |
| Arthritis, n (%) | 8 (80) | 0 |
| Large vessels vasculopathy, n (%) | 3 (30) | 0 |
