# Supplementary material for: Investigation of Newly Diagnosed Drug-Naive Patients with Systemic Autoimmune Diseases Revealed the Cleaved Peptide Tyrosine Tyrosine (PYY 3-36) as a Specific Plasma Biomarker of Rheumatoid Arthritis
Source: Mediators Inflamm. 2021 Jun 17;2021:5523582. doi: 10.1155/2021/5523582 (PMC8240466; doi:10.1155/2021/5523582)
Supplement: Supplementary 4 — Supplementary Table 4: the list of the proteins measured in the plasma of the human subjects enrolled in the study. The lower range of the detection corresponds to the threshold of the sensitivity of the assay. The Procarta Plex™ panel was designed by the authors, and the assay was loaded on a MAGPIX Luminex instrument. [file 5523582.f4.docx]

| **Short name of the protein** | **Full name of the protein** | **Aternative name of the protein** | **Gene name** | **UniProtPK ID** | **Detection range pg/ml** |
| --- | --- | --- | --- | --- | --- |
| SDF-1a | Stromal cell-derived factor 1 | C-X-C motif chemokine 12 | CXCL12 | P48061 | 6.33 - 120,139 |
| GITRL | Tumor necrosis factor ligand superfamily member 18 | Glucocorticoid-induced TNF-related ligand | TNFSF18 | Q9UNG2 | 27.74 - 283,018 |
| IL-1b | Interleukin-1 beta | Catabolin | IL1B | P01584 | 0.66 - 24,809 |
| IL-2 | Interleukin-2 | T-cell growth factor | IL2 | P60568 | 2.65 - 50,509 |
| IL-4 | Interleukin-4 | B-cell stimulatory factor 1 | IL4 | P05112 | 3.88 - 82,410 |
| IL-5 | Interleukin-5 | B-cell differentiation factor I | IL5 | P05113 | 6.32 - 38,16 |
| IL-33 | Interleukin-33 | Nuclear factor from high endothelial venule | IL33 | O95760 | 0.75 - 36,844 |
| IL-10 | Interleukin-10 | Cytokine synthesis inhibitory factor | IL10 | P22301 | 0.52 - 29,576 |
| Insulin | - | - | INS | P01308 | 48.81 - 1,614,592 |
| PYY | Peptide YY | Peptide tyrosine tyrosine | PYY | P10082 | 9.81 - 48,392 |
| CCL22 | C-C motif chemokine 22 | Macrophage-derived chemokine (MDC) | CCL22 | O00626 | 6.88 - 15,080 |
| IL-13 | Interleukin-13 | Allergic rhinhitis (ALRH) protein, bronchial hyperresponsiveness 1 (BHR1) protein, | IL13 | P35225 | 1.31 - 88,239 |
| IL-17A | Interleukin-17A | Cytotoxic T-lymphocyte-associated antigen 8 | IL17A | Q16552 | 0.68 - 17,761 |
| Gal-3 | Galectin-3 | Galactose-specific lectin 3 | LGALS3 | P17931 | 209.65 - 3,004,308 |
| FKN | Fractalkine | chemokine (C-X3-C motif) ligand 1 (CX3CL1) | CX3CL1 | P78423 | 0.61 - 5,900 |
| IFN-γ | Interferon gamma | IFNG, IFN-gamma | IFNG | P01579 | 5.67 - 89,348 |
| GM-CSF | Granulocyte-macrophage colony-stimulating factor | colony-stimulating factor 2 | CSF2 | P04141 | 6.02 - 114,860 |
| Leptin | - | Obese protein | LEP | P41159 | 4.86 - 155,005 |
| MMP-12 | Macrophage metalloelastase | Matrix metalloproteinase-12 | MMP12 | P39900 | 1.82 - 38,417 |
| NTproBNP | Natriuretic peptides B | N-terminal pro b-type natriuretic peptide | NPPB | P16860 | 1.69 - 174,28 |
| MCP-1 | C-C motif chemokine 2 | Monocyte chemoattractant protein 1 | CCL2 | P13500 | 1.9 - 26,623 |
| APRIL | A proliferation-inducing ligand | tumor necrosis factor ligand superfamily member 13 (TNFSF13) | N/A | Q6U6I7 | 20.64 - 972,758 |
| TNFRSF6 | Tumor necrosis factor receptor superfamily member 6 | Apo-1 antigen, Apoptosis-mediating surface antigen FAS, CD95 | FAS | P25445 | 11.23 - 685,148 |
| BDNF | Brain-derived neurotrophic factor | Abrineurin | BDNF | P23560 | 0.68 - 25,573 |
| BMP-9 | Growth/differentiation factor 2 | Bone morphogenetic protein 9 (BMP-9) | GDF2 | Q9UK05 | 2.25 - 66,256 |
| IL-12p40 | Interleukin-12 subunit beta | Cytotoxic lymphocyte maturation factor 40 kDa subunit | IL12B | P29460 | 0.63 - 7,067 |
| BAFF | Tumor necrosis factor ligand superfamily member 13B | B-cell- activating factor (BAFF) | TNFSF13B | Q9Y275 | 0.59 - 46,837 |
| M-CSF | Macrophage colony-stimulating factor 1 | Lanimostim, CSF-1 | CSF1 | P09603 | 7.71 - 110,910 |
| Survivin | Baculoviral IAP repeat-containing protein 5 | Apoptosis inhibitor survivin | BIRC5 | O15392 | 37.19 - 1,116,070 |
| CD40-ligand | CD40 ligand | Tumor necrosis factor ligand superfamily member 5 | CD40LG | P29965 | 0.81 - 28,729 |
| **Supplementary Table 4.** The list of the proteins measured in the plasma of the human subjects enrolled in the study. | | |  |  |  |
